# Editorial: Biodegradation of plastics and their additives

**DOI:** 10.3389/fmicb.2026.2012286

**Published:** 2026-10-14

**Authors:** Cristiano Varrone, Memory Tekere, Jinwei Zhang, Alfonso Olaya-Abril

**Affiliations:** 1 Department of Chemistry and BioScience, Aalborg University, Aalborg, Denmark; 2 Department of Environmental Sciences, College of Agriculture and Environmental Sciences, University of South Africa (UNISA), Roodepoort, South Africa; 3 State Key Laboratory of Chemical Biology, Research Center of Chemical Kinomics, Shanghai Institute of Organic Chemistry, Chinese Academy of Sciences, Shanghai, China; 4 Department of Biochemistry and Molecular Biology, University of Córdoba, Córdoba, Spain

**Keywords:** biocatalysis, biodegradation, bioprospecting, microbial consortia, plastics, plastics additives, synthetic biology

Plastic pollution ranks among the most pressing environmental challenges of our time, with polymers such as polyethylene (PE), polyethylene terephthalate (PET), polystyrene (PS) and their chemical additives persisting across terrestrial, marine and even polar ecosystems. Microbial biodegradation offers a biologically grounded route toward mitigating this burden, but progress depends on continually expanding the catalog of organisms, enzymes and metabolic strategies capable of attacking these recalcitrant materials. This Research Topic gathers ten contributions that, together, illustrate both how far the field has come and where its next challenges lie.

## Mining underexplored niches for novel degraders

A recurring strategy across this Research Topic is bioprospecting in environments where selective pressure for plastic degradation is plausible but underexploited. An et al. isolated *Papiliotrema laurentii* from locust frass and characterized its broad-spectrum activity against aliphatic and aliphatic-aromatic polyesters. Berríos-Farías et al. mined Antarctic soils and rhizospheres by metagenomics to identify candidate PET hydrolases, extending the search for degrading enzymes to psychrophilic communities. Ruginescu et al. characterized R75estA, a halophilic polyester-degrading enzyme from a marine bacterium, adding to the growing evidence that extremophiles harbor biocatalysts with distinct operational advantages. Insect guts proved similarly fruitful: Biełło et al. examined the response of the *Tenebrio molitor* gut microbiota to LDPE and PET, while de Souza et al. showed that *Paenibacillus lautus*, isolated from the *Sphenophorus levis* gut, induces structural and physicochemical changes on polystyrene. Together, these five studies reinforce insect and extreme-environment microbiomes as reservoirs of biocatalytic diversity still far from exhausted.

## From discovery to mechanism

Several contributions moved beyond isolation toward mechanistic characterization. Dudziak et al. applied proteomics to *Lysinibacillus* to reveal early-stage PET biodegradation potential, and Biełło et al. combined metagenomic and proteomic evidence to link predicted functions with proteins detected in the gut community. These omics-driven approaches, together with the enzymatic characterization by Ruginescu et al., exemplify a shift in the field's focus toward understanding the enzymatic machinery involved, moving beyond merely identifying which organisms degrade plastics. Such mechanistic understanding is essential for moving from enzyme discovery toward rational optimization and engineering of plastic-degrading biocatalysts. The next step, however, is not simply to find more enzymes, but to integrate enzymes, organisms and complementary functions into systems capable of addressing real materials and complex mixtures.

## From individual degraders to functional communities

Plastic waste further complicates this picture because real-world materials rarely consist of a single chemically uniform polymer. Additives, coatings, pigments and weathering-derived products create heterogeneous substrates that may require different catalytic and metabolic capabilities. This chemical heterogeneity provides a rationale for moving from the search for individual “plastic degraders” toward communities capable of complementary transformations. Complex polymeric substrates typically require sequential catalytic steps, including surface colonization, extracellular depolymerization, and uptake and intracellular catabolism of heterogeneous monomers and additive fragments, that few single strains perform efficiently end-to-end. This mirrors established principles for other recalcitrant substrates, such as lignocellulose, where synergistic, division-of-labor consortia are well recognized (Figure 1). Bertoldi et al. provide direct experimental support for this view in their study of cross-feeding during the degradation of phthalate ester plasticizers by a bacterial consortium—one of the few contributions to this Research Topic to explicitly address plastic additives rather than the polymer backbone itself. Narayanan formalizes this logic theoretically, proposing a systems-biology framework in which engineered microbial consortia degrade mixed plastic streams and convert them into fertilizer-grade compounds, pointing toward valorization rather than mere mineralization as an end goal. Taken together with the broader literature, these findings support a shift from viewing microbial consortia as a specialized strategy toward considering functional community design as a central approach for tackling plastic waste.

**Figure 1:**
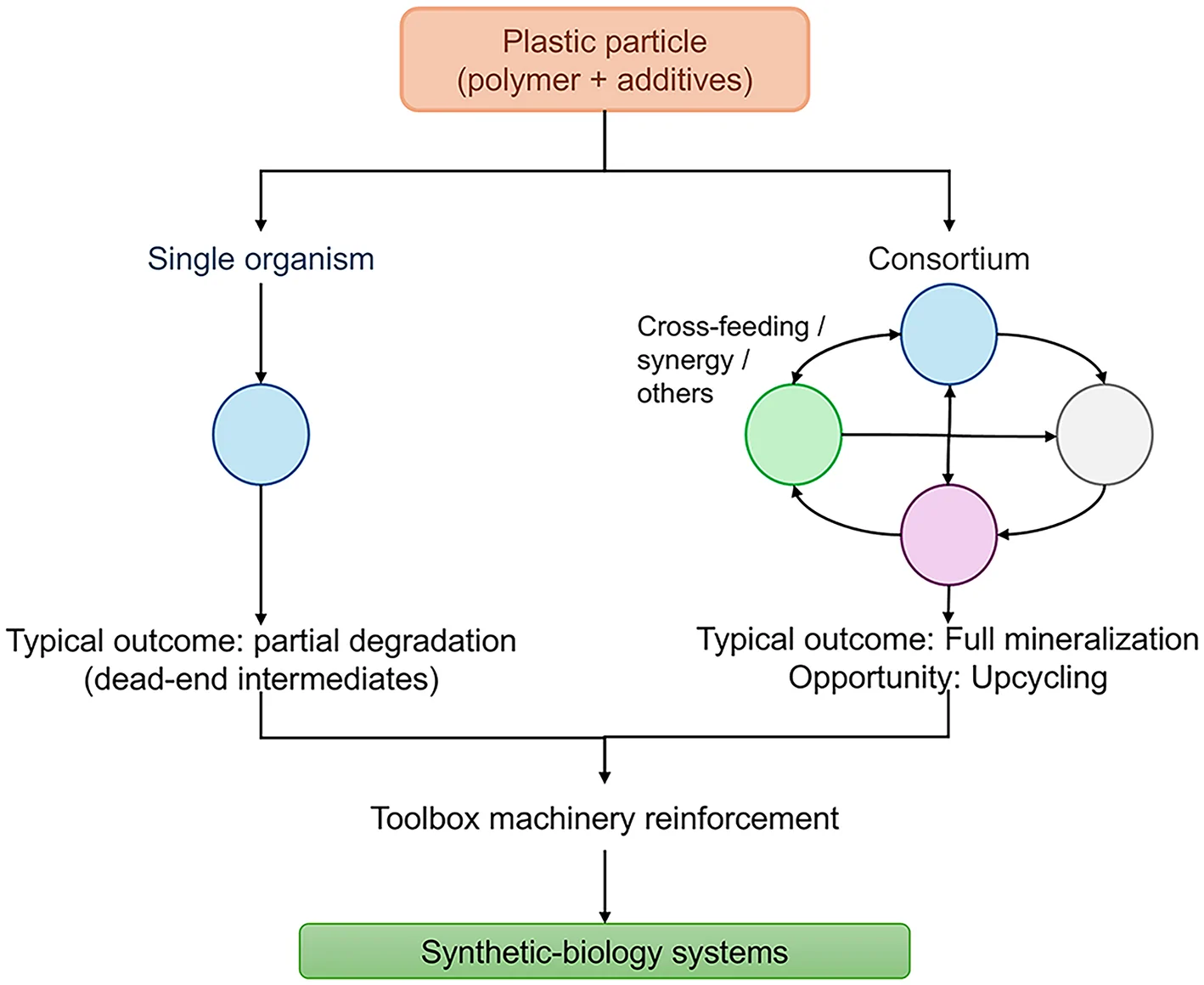
Conceptual comparison of single-strain versus consortium-based biodegradation of a complex plastic substrate containing polymer backbone and additives.

## Expanding the toolkit and the range of agents

Two further contributions broaden the scope of usable strategies. Zeng et al. combined UV irradiation and acridine orange mutagenesis to enhance polyethylene biodegradation by *Sphingobacterium prati* BS2, demonstrating that classical strain-improvement approaches remain a viable complement to bioprospecting. Besler et al. extended the range of degrading agents beyond bacteria and fungi altogether, showing that the microalgae *Scenedesmus dimorphus* and *Chlorella vulgaris* cause surface bio-erosion and structural alteration of colored and agricultural plastics. This serves as a reminder that photosynthetic microorganisms deserve more systematic attention in this field.

## Gaps and outlook

Taken together, these ten studies confirm steady progress in identifying novel degraders and elucidating their molecular toolkits, particularly for PET, PE, and polyester-type polymers. Two gaps stand out. First, despite the Topic's explicit focus on “plastics and their additives,” only Bertoldi et al. directly addressed additive degradation; plasticizers, stabilizers and colorants remain comparatively neglected relative to the polymer backbone, and closing this gap should be a priority for future Topics. Second, while Bertoldi et al. and Narayanan point toward consortium- and systems-level approaches, most contributions still characterize single isolates; translating this growing catalog of individual degraders into rationally designed, robust multispecies communities—and eventually into scalable bioremediation or valorization processes—remains the central challenge ahead. Realizing this potential will also depend on scaling discovery itself: ultra-high-throughput platforms capable of screening thousands of enzymes, strains or mutants, coupled with machine learning-guided optimization, are gaining traction in the field and could substantially accelerate the transition from individually characterized biocatalysts to deployable degradation systems.

An important challenge is therefore to distinguish surface colonization and physicochemical alteration from genuine biodegradation, supported by evidence of depolymerization, assimilation and ultimately carbon flux through the degradation pathway.

Collectively, these studies illustrate that the search for plastic-degrading microorganisms is no longer reduced to environments chronically exposed to plastics. Instead, relevant biocatalytic functions can emerge from ecological niches selected for other physicochemical pressures, suggesting that future bioprospecting may benefit from treating environmental extremes and host-associated microbiomes as sources of biochemical novelty.

We thank all authors and reviewers for their contributions to this Research Topic, and hope it stimulates further research bridging bioprospecting, mechanistic characterization and consortium engineering in pursuit of sustainable solutions to plastic pollution.

